# Data for crystallisation, dissolution and saturation temperatures of the ternary system: Hexadecane and octadecane representative in fuel solvents

**DOI:** 10.1016/j.dib.2018.05.136

**Published:** 2018-05-31

**Authors:** Xue Tang, Peter L. Kaskiewicz, Diana M. Camacho Corzo, Xiaojun Lai, Kevin J. Roberts, Peter Dowding, Iain More

**Affiliations:** a School of Chemical and Process Engineering, University of Leeds, Leeds, UK; b Infineum UK Ltd., Milton Hill Business and Technology Centre, Abingdon, UK

## Abstract

The data presented in this article relates to the crystallisation of hexadecane (C_16_H_34_) and octadecane (C_18_H_38_), being the predominant alkanes present in hydrotreated vegetable oil (HVO), from solvents representative of fuel (dodecane, toluene and kerosene). Data was collected for eleven C_16_H_34_/C_18_H_38_ compositions for each solvent used. Raw crystallisation and dissolution data is provided over a range of solution concentrations and cooling rates used under a poly-thermal crystallisation methodology. Equilibrium saturation temperature data is also presented for each composition, concentration and solvent system, indicating the trend in solubility for each solution.

**Specifications Table**

**Table:** 

Subject area	*Chemical Engineering*
More specific subject area	*Crystallisation and Fuel*
Type of data	*Tables and figures*
How data was acquired	*In this study the data was collected from crystallisation experiments using the Avantium Crystal16 apparatus.*
Data format	*Analysed*
Experimental factors	*In all experiments the solutions were homogenised through heat and agitation before crystallisation took place. Each experimental run was repeated five times.*
Experimental features	*Crystallisation studies were performed to acquire data concerning the crystallisation and dissolution temperatures in order to calculate equilibrium saturation temperatures and solubility factors.*
Data source location	*University of Leeds, Leeds, West Yorkshire, United Kingdom*
Data accessibility	*Data is with this article*
Related research article	*X. Tang, P.L. Kaskiewicz, D.M. Camacho Corzo, X. Lai, K.J. Roberts, P. Dowding and I. More. Solubility and Crystallisability of the Ternary System: Hexadecane and Octadecane Representative in Fuel Solvents.* Fuel. 2018;226(2018):665-674.

**Value of the data**

• Crystallisation of binary C_16_H_34_/C_18_H_38_ alkane mixtures in HVO fuel under cold weather conditions is a current technological problem and isn’t well understood, therefore, data collected in relation to this provides an insight into the behaviour.
• Crystallisation of binary C_16_H_34_/C_18_H_38_ alkane mixtures is also important in terms of the blending of HVO derived biofuel into conventional diesel fuel.
• The data enables key nucleation parameters to be determined as well as the mechanism of nucleation, leading to a greater understanding of how crystallisation processes in HVO fuels occur.
• Future work on further understanding and potentially controlling alkane crystallisation in HVO fuel with a focus on additive selection can be built from this data.
• The current global focus on ‘cleaner’ transportation fuel in order to reduce environment global warming impact means this data is very relevant towards current and future advancements.

## 1 Data

Average crystallisation (*T*_c_) and dissolution (*T*_diss_) temperatures for each of the eleven C_16_H_34_/C_18_H_38_ compositions at each cooling/heating rate (*q*) used for the crystallisation experiments and each solution concentration are shown in Table 1, Table 2, Table 3, for the solvents dodecane, kerosene and toluene, respectively. The standard deviation (std) of the data is also displayed. The data provided for each solvent used highlights the influence that the solute composition, solute concentration and cooling rate has upon the crystallisation behaviour of C_16_H_34_/C_18_H_38_ binary mixtures.

**Table 1:** Average temperatures of crystallisation (*T*_c_) and dissolution (*T*_diss_) as a function of cooling rates and binary mixture compositions of C_16_H_34_/C_18_H_38_ in dodecane solution at concentrations of 192, 231, 269, 308 g/l. STD: standard deviation of the measured *T*_c_ and *T*_diss_ from 5 recycles of repeats. The data highlights the influence of solute composition, solute concentration and cooling rate on the resultant crystallisation behaviour.

**x C18 / q(◦C/min)**	C16:C18 (100:0)				C16:C18 (90:10)				C16:C18 (80:20)				C16:C18 (70:30)				
	$T_{c}$**(°C)**	**STD (°C)**	$T_{\mathrm{diss}}$**(°C)**	**STD (°C)**	$T_{c}$**(°C)**	**STD (°C)**	$T_{\mathrm{diss}}$**(°C)**	**STD (°C)**	$T_{c}$**(°C)**	**STD (°C)**	$T_{\mathrm{diss}}$**(°C)**	**STD (°C)**	$T_{c}$**(°C)**	**STD (°C)**	$T_{\mathrm{diss}}$**(°C)**	**STD (°C)**	
	192 g/l				192 g/l				192 g/l				192 g/l				
0.25	−8.88	0.51	−3.7	0.07	−13.26	0.09	−7.02	0.08	−12.74	0.05	−7.7	0	−11.93	0.04	−7.68	0.09	
1	−10.44	0.41	−0.2	0.21	−14.5	0.07	−3.38	0.13	−14.08	0.04	−4.78	0.19	−12.58	0.08	−3.58	0.19	
2	−11.48	0.61	3.36	0.4	−14.72	0.08	2.38	0.19	−14.8	0	−1.18	0.04	−13.42	0.08	−0.14	0.36	
3.2	−12.68	0.25	8.2	0.25	−14.88	0.04	6.36	0.32	−14.78	0.04	4.12	0.16	−14.06	0.09	3.22	0.5	
	231 g/l					231 g/l			231 g/l				231 g/l				
0.25	−6.26	0.67	−1.72	0.04	−11.12	0.11	−4.68	0.08	−10.44	0.05	−7.2	0	−9.63	0.04	−7.3	0.04	
1	−7.16	0.25	1.04	0.13	−12.46	0.05	−1.46	0.09	−11.68	0.04	−4.46	0.21	−10.48	0.11	−3.42	0.08	
2	−8.02	0.2	4.32	0.24	−12.98	0.08	3.78	0.19	−12.56	0.05	−0.66	0.15	−11.18	0.04	0.38	0.18	
3.2	−9.08	0.26	9.54	0.59	−13.64	0.11	7.12	0.45	−12.82	0.11	4.72	0.24	−12.2	0	3.96	0.54	
	269 g/l					269 g/l			269 g/l				269 g/l				
0.25	−3.94	0.44	−0.18	0.04	−9.42	0.08	−2.84	0.05	−8.86	0.05	−6.04	0.05	−7.8	0.07	−6.08	0.04	
1	−4.64	0.62	2.44	0.48	−10.34	0.09	−0.24	0.05	−10.16	0.05	−3.56	0.13	−8.54	0.05	−3.72	0.08	
2	−6.18	0.33	7.28	0.18	−10.48	0.18	4.46	0.09	−11.16	0.09	0.54	0.25	−9.2	0.07	0.2	0.07	
3.2	−6.92	0.37	10.6	0.58	−11.98	0.04	9.42	0.2	−11.14	0.13	4.96	0.13	−10.3	0.21	3.04	0.57	
	308 g/l					308 g/l			308 g/l				308 g/l				
0.25	−2.74	0.56	1.04	0.05	−8.3	0.07	−1.48	0.08	−7.24	0.05	−4.48	0.2	−6.54	0.05	−5.22	0.08	
1	−3.54	0.47	3.02	0.11	−9.12	0.04	0.36	0.09	−8.54	0.05	−3.12	0.11	−7.4	0.1	−3.38	0.08	
2	−5.02	0.22	8.08	0.08	−9.52	0.08	4.94	0.09	−9.48	0.11	0.98	0.18	−7.9	0.07	−0.62	0.13	
3.2	−6.12	0.54	11.42	0.36	−10.54	0.13	10.04	0.09	−9.76	0.15	5.3	0.26	−9.3	0	2.38	0.22	
	C16:C18 (60:40)					C16:C18 (50:50)			C16:C18 (40:60)				C16:C18 (30:70)				
	192 g/l					192 g/l			192 g/l				192 g/l				
0.25	−10.34	0.13	−3.62	0.04	−6.46	0.32	−0.6	0.07	−3.14	0.13	1.28	0.04	−0.58	0.39	3.1	0	
1	−11.56	0.05	−2.88	0.13	−7.64	0.18	0.72	0.11	−4.22	0.19	2.34	0.05	−1.72	0.78	3.7	0.1	
2	−12.44	0.13	0.22	0.08	−8.88	0.39	2.5	0.2	−5.12	0.15	6.44	0.22	−2.1	0.14	7.18	0.11	
3.2	−13.18	0.13	4.58	0.27	−10.54	0.09	7.06	0.25	−7.18	0.04	12.04	0.18	−3.96	0.36	11.38	0.29	
	231 g/l					231 g/l			231 g/l				231 g/l				
0.25	−8.38	0.08	−1.8	0	−4.82	0.13	1.24	0.05	−1.66	0.23	2.9	0	0.6	0.27	4.8	0.07	
1	−9.42	0.11	−2.06	0.05	−5.76	0.26	2.22	0.15	−2.56	0.09	3.58	0.04	0.06	0.55	5.54	0.09	
2	−10.2	0.07	0.34	0.11	−7.34	0.18	4.98	0.18	−3.7	0.14	6.04	0.09	−0.58	0.08	8.42	0.29	
3.2	−10.32	0.22	3.64	0.15	−8.14	0.11	8.56	0.23	−4.46	0.17	10.02	0.27	−1.86	0.54	12.84	0.49	
	269 g/l					269 g/l			269 g/l				269 g/l				
0.25	−6.64	0.09	−0.6	0.07	−3.48	0.39	2.72	0.04	−0.1	0.16	4.5	0	2.68	0.28	6.2	0	
1	−7.6	0	−1.1	0.12	−4.38	0.11	3.34	0.09	−1.1	0.1	5.16	0.09	1.7	0.31	6.7	0.07	
2	−8	0	0.3	0.07	−5.76	0.17	6.02	0.08	−2.5	0.12	7.96	0.23	0.76	0.11	9.38	0.11	
3.2	−8.88	0.04	4.72	0.2	−6.48	0.19	8.88	0.2	−3.22	0.19	12.2	0.39	−0.06	0.15	12.68	0.36	
	308 g/l					308 g/l			308 g/l				308 g/l				
0.25	−5.24	0.05	0.34	0.05	−2.42	0.26	3.76	0.05	1.08	0.08	5.62	0.04	3.76	0.11	7.48	0.04	
1	−6.2	0	−0.42	0.08	−3.52	0.08	4.6	0.1	−0.06	0.15	6.18	0.04	2.42	0.4	8.42	0.04	
2	−7.12	0.11	0.58	0.04	−4.5	0.14	6.74	0.05	−0.96	0.09	7.62	0.22	1.88	0.04	10.4	0.28	
3.2	−7.32	0.16	3.1	0.19	−5.06	0.09	9.26	0.25	−2.52	0.2	13.02	0.11	1.02	0.22	13.7	0.21	
	C16:C18 (20:80)					C16:C18 (10:90)			C16:C18 (0:100)								
	192 g/l					192 g/l			192 g/l								
0.25	2	0.1	4.28	0.04	3.66	0.11	5.54	0.05	4.56	0.35	6.8	0					
1	1.12	0.08	5.18	0.13	2.94	0.29	6.8	0.1	3.16	0.55	7.8	0					
2	−0.32	0.27	8.4	0.12	2.28	0.18	9.7	0.14	1.88	0.26	11.9	0.24					
3.2	−1.22	0.18	13.38	0.18	0.82	0.38	14.08	0.37	1.38	0.52	14.86	0.28					
	231 g/l					231 g/l			231 g/l								
0.25	3.86	0.05	6.08	0.04	4.9	0.26	7.24	0.05	6.6	0.29	8.42	0.04					
1	2.94	0.09	7.16	0.09	4.7	0.16	8.42	0.04	5.16	0.51	9.68	0.11					
2	1.88	0.16	9.72	0.16	4.04	0.17	10.64	0.26	4.2	0.2	12.46	0.27					
3.2	1.02	0.18	13.66	0.76	3.18	0.26	14.62	0.25	3.38	0.54	16.02	0.49					
	269 g/l					269 g/l			269 g/l								
0.25	5.36	0.09	7.54	0.05	6.84	0.11	8.7	0	8.1	0.23	10	0.07					
1	4.38	0.04	8.64	0.13	5.78	0.2	10.08	0.04	6.66	0.21	11.18	0.15					
2	3	0.35	10.2	0.22	4.94	0.09	11.74	0.26	5.54	0.29	13.68	0.16					
3.2	2.5	0.16	15.64	0.26	4.6	0.14	16.28	0.23	5.42	0.31	17.1	0.34					
	308 g/l					308 g/l			308 g/l								
0.25	6.42	0.04	8.72	0.04	7.74	0.36	10.04	0.05	9.5	0.1	11.16	0.05					
1	5.46	0.09	9.78	0.18	6.3	0.42	11.44	0.11	8.3	0.3	12.46	0.24					
2	4.4	0	11.24	0.11	5.66	0.13	14.08	0.22	7.3	0.46	14.98	0.15					
3.2	3.96	0.15	15.16	0.35	5.3	0.24	16.94	0.33	6.64	0.36	18.68	0.28

**Table 2:** Average temperatures of crystallisation (*T*_c_) and dissolution (*T*_diss_) as a function of cooling rates and binary mixture compositions of C_16_H_34_/C_18_H_38_ in kerosene solution at concentrations of 231, 269, 308, 350 g/l. STD: standard deviation of the measured *T*_c_ and *T*_diss_ from 5 recycles of repeats. The data highlights the influence of solute composition, solute concentration and cooling rate on the resultant crystallisation behaviour.

**x C18 / q(◦C/min)**	C16:C18 (100:0)				C16:C18 (90:10)				C16:C18 (80:20)				C16:C18 (70:30)			
	$T_{c}$**(°C)**	**STD (°C)**	$T_{\mathrm{diss}}$**(°C)**	**STD (°C)**	$T_{c}$**(°C)**	**STD (°C)**	$T_{\mathrm{diss}}$**(°C)**	**STD (°C)**	$T_{c}$**(°C)**	**STD (°C)**	$T_{\mathrm{diss}}$**(°C)**	**STD (°C)**	$T_{c}$**(°C)**	**STD (°C)**	$T_{\mathrm{diss}}$**(°C)**	**STD (°C)**
	231 g/l				231 g/l				231 g/l				231 g/l			
0.25	−5.98	0.26	−2.68	0.04	−11.62	0.31	−5.78	0.04	−12.70	0.07	−8.14	0.05	−11.40	0.10	−9.06	0.11
1	−6.54	0.42	−1.46	0.05	−12.14	0.18	−5.34	0.05	−13.18	0.04	−7.32	0.04	−12.16	0.05	−8.72	0.04
2	−6.78	0.40	0.52	0.08	−13.24	0.18	−4.00	0.14	−13.82	0.04	−6.02	0.08	−12.98	0.22	−7.62	0.08
3.2	−8.46	0.49	3.26	0.37	−14.46	0.05	−2.56	0.34	−14.52	0.04	−4.58	0.18	−14.34	0.05	−7.00	0.24
	269 g/l				269 g/l				269 g/l				269 g/l			
0.25	−4.74	0.29	−1.22	0.04	−9.76	0.34	−4.00	0.14	−10.64	0.05	−5.98	0.04	−9.44	0.05	−7.40	0.07
1	−4.46	0.56	0.04	0.05	−10.66	0.15	−3.64	0.05	−11.24	0.05	−5.52	0.13	−10.24	0.09	−6.76	0.11
2	−5.16	0.66	2.12	0.26	−11.44	0.22	−2.22	0.30	−11.86	0.15	−4.18	0.13	−11.02	0.11	−5.62	0.04
3.2	−6.14	0.25	4.68	0.04	−12.50	0.00	−0.52	0.37	−13.04	0.13	−2.72	0.04	−12.24	0.22	−4.52	0.11
	308 g/l				308 g/l				308 g/l				308 g/l			
0.25	−2.82	0.30	0.12	0.04	−8.42	0.26	−2.78	0.04	−9.10	0.00	−4.52	0.04	−7.88	0.04	−5.70	0.00
1	−3.52	0.35	1.68	0.11	−9.24	0.05	−2.06	0.05	−9.58	0.04	−4.02	0.04	−8.68	0.08	−5.02	0.04
2	−4.54	0.63	3.70	0.12	−10.06	0.13	−0.66	0.09	−10.34	0.11	−2.64	0.11	−9.28	0.15	−3.50	0.12
3.2	−4.98	0.29	6.60	0.14	−12.50	0.00	1.42	0.20	−11.10	0.19	−1.44	0.17	−10.20	0.00	−2.10	0.17
	350 g/l				350 g/l				350 g/l				350 g/l			
0.25	−1.92	0.43	1.16	0.05	−7.06	0.24	−1.68	0.04	−7.82	0.04	−3.56	0.05	−6.46	0.05	−4.54	0.05
1	−2.18	0.60	2.66	0.15	−8.12	0.04	−0.92	0.04	−8.52	0.13	−3.06	0.09	−7.46	0.05	−3.92	0.08
2	−3.20	0.47	5.36	0.09	−8.90	0.07	0.78	0.16	−9.14	0.05	−1.52	0.13	−8.10	0.14	−1.98	0.18
3.2	−3.38	0.29	7.24	0.26	−9.88	0.04	2.54	0.26	−10.02	0.16	0.24	0.09	−9.12	0.04	0.02	0.19

	C16:C18 (60:40)				C16:C18 (50:50)				C16:C18 (40:60)				C16:C18 (30:70)			
	231 g/l				231 g/l				231 g/l				231 g/l			
0.25	−9.62	0.04	−3.80	0.00	−5.94	0.18	−0.56	0.09	−3.30	0.29	1.60	0.00	−0.96	0.24	3.18	0.04
1	−10.38	0.04	−4.60	0.12	−7.24	0.15	−0.18	0.08	−4.22	0.29	2.20	0.10	−1.88	0.23	3.96	0.09
2	−11.48	0.08	−5.00	0.10	−8.22	0.04	0.84	0.09	−5.42	0.20	3.40	0.10	−2.60	0.27	5.04	0.05
3.2	−12.38	0.16	−4.76	0.26	−9.46	0.09	2.14	0.09	−6.60	0.23	5.00	0.14	−3.94	0.22	7.20	0.31
	269 g/l				269 g/l				269 g/l				269 g/l			
0.25	−7.76	0.11	−2.80	0.10	−4.56	0.13	0.90	0.00	−1.82	0.28	3.14	0.05	0.46	0.09	4.54	0.09
1	−8.52	0.08	−5.06	0.11	−5.66	0.13	1.36	0.05	−2.40	0.10	3.76	0.09	−0.44	0.17	5.42	0.04
2	−9.62	0.04	−4.10	0.19	−6.68	0.13	2.52	0.13	−3.54	0.18	4.92	0.11	−1.50	0.20	6.58	0.13
3.2	−10.48	0.08	−2.48	0.68	−7.70	0.12	3.78	0.11	−4.54	0.15	6.48	0.29	−2.46	0.25	8.72	0.29
	308 g/l				308 g/l				308 g/l				308 g/l			
0.25	−6.28	0.08	−1.64	0.31	−3.58	0.16	2.10	0.00	−0.74	0.35	4.28	0.04	1.90	0.31	5.92	0.04
1	−7.20	0.07	−2.38	0.16	−4.92	0.18	2.66	0.15	−1.50	0.25	4.88	0.04	1.28	0.18	6.84	0.13
2	−7.90	0.07	−3.02	0.15	−5.54	0.09	3.66	0.18	−2.50	0.30	6.24	0.11	0.44	0.23	8.16	0.11
3.2	−8.90	0.00	−1.28	0.13	−6.64	0.05	5.08	0.04	−3.46	0.09	8.44	0.15	−0.70	0.32	10.48	0.36
	350 g/l				350 g/l				350 g/l				350 g/l			
0.25	−4.90	0.07	−0.28	0.04	−2.56	0.11	3.34	0.05	0.44	0.21	5.38	0.04	3.36	0.11	7.10	0.00
1	−5.90	0.00	−1.04	0.09	−3.90	0.14	3.94	0.09	−0.68	0.08	6.20	0.00	2.20	0.25	8.14	0.09
2	−6.60	0.00	−0.30	0.10	−4.76	0.11	5.16	0.09	−1.56	0.23	7.60	0.12	1.20	0.25	9.76	0.17
3.2	−7.36	0.22	0.52	0.24	−5.66	0.13	6.68	0.08	−2.38	0.16	9.02	0.16	0.28	0.11	11.70	0.14
	C16:C18 (20:80)				C16:C18 (10:90)				C16:C18 (0:100)							
	231 g/l				231 g/l				231 g/l							
0.25	1.02	0.63	4.64	0.05	3.22	0.26	6.00	0.00	4.94	0.30	7.42	0.04				
1	0.50	0.35	5.68	0.08	2.78	0.11	7.30	0.12	4.18	0.27	8.68	0.04				
2	0.00	0.41	6.98	0.16	2.00	0.37	8.64	0.29	3.20	0.24	10.52	0.13				
3.2	−1.18	0.22	9.50	0.31	0.70	0.35	10.96	0.42	2.46	0.43	13.00	0.32				
	269 g/l				269 g/l				269 g/l							
0.25	3.26	0.09	6.20	0.00	5.26	0.15	7.54	0.05	6.30	0.46	8.88	0.04				
1	2.30	0.23	7.32	0.04	4.46	0.18	8.96	0.09	5.70	0.40	10.42	0.11				
2	1.44	0.30	8.90	0.12	3.38	0.13	11.28	0.08	5.04	0.42	12.38	0.22				
3.2	0.24	0.38	11.50	0.30	2.48	0.22	12.84	0.33	4.24	0.47	14.64	0.15				
	308 g/l				308 g/l				308 g/l							
0.25	4.14	0.28	7.48	0.04	6.30	0.20	8.70	0.00	7.78	0.19	10.10	0.07				
1	3.64	0.23	8.70	0.00	5.80	0.21	10.32	0.13	7.10	0.52	11.56	0.09				
2	2.74	0.32	10.16	0.18	4.56	0.27	12.36	0.18	6.24	0.30	13.70	0.33				
3.2	2.32	0.04	12.92	0.22	3.20	0.32	14.54	0.18	5.56	0.25	16.46	0.13				
	350 g/l				350 g/l				350 g/l							
0.25	5.40	0.44	8.68	0.04	7.60	0.31	9.92	0.04	9.20	0.28	11.36	0.05				
1	4.82	0.08	10.02	0.04	6.90	0.14	11.60	0.00	8.38	0.16	12.80	0.12				
2	3.68	0.40	11.56	0.17	6.16	0.15	13.10	0.07	7.26	0.75	15.46	0.13				
3.2	2.84	0.48	14.18	0.25	5.38	0.16	15.46	0.26	6.98	0.16	18.18	0.16

**Table 3:** Average temperatures of crystallisation (*T*_c_) and dissolution (*T*_diss_) as a function of cooling rates and binary mixture compositions of C_16_H_34_/C_18_H_38_ in toluene solution at concentrations of 300, 350, 400, 350 g/l. STD: standard deviation of the measured *T*_c_ and *T*_diss_ from 5 recycles of repeats. The data highlights the influence of solute composition, solute concentration and cooling rate on the resultant crystallisation behaviour.

**x C18 / q(◦C/min)**	C16:C18 (100:0)				C16:C18 (90:10)				C16:C18 (80:20)				C16:C18 (70:30)			
	$T_{c}$**(°C)**	**STD (°C)**	$T_{\mathrm{diss}}$**(°C)**	**STD (°C)**	$T_{c}$**(°C)**	**STD (°C)**	$T_{\mathrm{diss}}$**(°C)**	**STD (°C)**	$T_{c}$**(°C)**	**STD (°C)**	$T_{\mathrm{diss}}$**(°C)**	**STD (°C)**	$T_{c}$**(°C)**	**STD (°C)**	$T_{\mathrm{diss}}$**(°C)**	**STD (°C)**
	300 g/l				300 g/l				300 g/l				300 g/l			
0.25	−5.83	0.21	−3.70	0.10	−10.52	0.24	−7.00	0.07	−12.72	0.08	−8.80	0.07	−11.97	0.06	−9.00	0.10
1	−6.10	0.64	−2.14	0.24	−11.70	0.16	−6.26	0.15	−13.40	0.00	−7.92	0.13	−12.62	0.08	−8.52	0.26
2	−6.74	0.43	−0.48	0.43	−12.14	0.29	−4.78	0.28	−14.12	0.19	−5.96	0.11	−12.94	0.11	−7.06	0.29
3.2	−8.20	0.63	2.16	0.29	−12.80	0.00	−2.72	0.11	−14.26	0.22	−4.26	0.15	−13.48	0.00	−5.26	0.05
	350 g/l				350 g/l				350 g/l				350 g/l			
0.25	−4.24	0.26	−2.30	0.00	−9.30	0.08	−5.74	0.05	−11.18	0.04	−7.46	0.05	−10.70	0.07	−7.88	0.04
1	−4.42	0.33	−0.80	0.10	−10.60	0.23	−4.80	0.19	−12.02	0.08	−6.20	0.00	−11.00	0.19	−6.94	0.05
2	−4.96	0.15	1.38	0.50	−11.02	0.16	−2.90	0.34	−12.20	0.12	−4.70	0.16	−11.48	0.13	−5.40	0.19
3.2	−6.02	0.34	3.34	0.38	−11.46	0.11	−0.34	0.22	−13.10	0.14	−2.44	0.23	−12.48	0.18	−3.52	0.04
	400 g/l				400 g/l				400 g/l				400 g/l			
0.25	−3.74	0.43	−1.62	0.26	−8.60	0.07	−4.46	0.05	−10.04	0.11	−6.46	0.05	−9.34	0.17	−6.90	0.07
1	−3.56	0.30	0.14	0.05	−9.66	0.13	−3.70	0.19	−10.70	0.10	−5.26	0.09	−10.36	0.21	−5.80	0.16
2	−4.14	0.11	1.82	0.53	−10.42	0.23	−1.74	0.34	−11.18	0.15	−3.36	0.17	−10.68	0.16	−3.96	0.05
3.2	−4.74	0.13	4.40	0.74	−10.66	0.05	0.58	0.22	−11.70	0.07	−1.28	0.16	−11.24	0.40	−2.44	0.22
	450 g/l				450 g/l				450 g/l				450 g/l			
0.25	−2.32	0.36	−0.46	0.05	−7.43	0.22	−3.62	0.08	−9.32	0.13	−5.54	0.15	−8.22	0.04	−6.08	0.04
1	−2.98	0.36	1.16	0.09	−9.02	0.41	−2.68	0.04	−10.10	0.07	−4.32	0.04	−9.12	0.08	−4.88	0.08
2	−3.06	0.18	2.82	0.08	−9.56	0.24	−1.00	0.14	−10.54	0.15	−2.50	0.07	−9.68	0.25	−2.56	0.13
3.2	−3.98	0.11	6.00	0.31	−10.16	0.26	2.12	0.43	−11.70	0.07	−0.28	0.16	−10.62	0.18	−0.88	0.23
	C16:C18 (60:40)				C16:C18 (50:50)				C16:C18 (40:60)				C16:C18 (30:70)			
	300 g/l				300 g/l				300 g/l				300 g/l			
0.25	−9.76	0.15	−4.48	0.04	−6.13	0.15	−1.03	0.06	−3.60	0.20	0.58	0.04	−0.96	0.29	2.46	0.09
1	−10.98	0.08	−6.66	0.05	−7.30	0.14	−0.90	0.07	−4.84	0.09	1.24	0.11	−1.94	0.30	3.34	0.05
2	−11.36	0.15	−5.70	0.14	−8.00	0.12	0.08	0.19	−5.08	0.22	2.54	0.15	−2.60	0.14	4.94	0.18
3.2	−11.90	0.00	−5.00	0.35	−9.38	0.08	1.24	0.15	−6.14	0.25	4.36	0.22	−2.92	0.22	6.80	0.26
	350 g/l				350 g/l				350 g/l				350 g/l			
0.25	−8.52	0.04	−3.36	0.05	−4.86	0.05	0.00	0.00	−2.46	0.29	1.86	0.05	0.25	0.10	3.42	0.11
1	−9.40	0.10	−4.46	0.09	−6.08	0.13	0.24	0.05	−3.44	0.09	2.36	0.05	−1.04	0.15	4.54	0.05
2	−9.80	0.07	−3.64	0.05	−6.92	0.04	1.42	0.13	−4.08	0.22	4.00	0.10	−1.66	0.15	5.90	0.10
3.2	−10.72	0.13	−1.74	0.17	−8.06	0.05	2.64	0.17	−4.82	0.30	5.54	0.21	−2.40	0.12	7.80	0.12
	400 g/l				400 g/l				400 g/l				400 g/l			
0.25	−7.52	0.08	−2.20	0.00	−4.18	0.13	0.90	0.00	−1.32	0.16	2.78	0.08	0.90	0.20	4.40	0.00
1	−8.34	0.05	−2.78	0.13	−5.24	0.15	1.34	0.05	−2.24	0.15	3.56	0.05	0.04	0.05	5.44	0.05
2	−8.90	0.09	−2.08	0.00	−5.72	0.13	2.36	0.09	−3.22	0.13	4.90	0.07	−0.62	0.22	7.34	0.21
3.2	−9.70	0.16	−0.92	0.22	−6.96	0.05	3.74	0.13	−4.12	0.16	6.82	0.34	−1.72	0.29	9.02	0.16
	450 g/l				450 g/l				450 g/l				450 g/l			
0.25	−6.82	0.10	−1.44	0.09	−3.32	0.15	1.64	0.05	−0.74	0.11	3.44	0.05	2.18	0.11	5.22	0.08
1	−7.64	0.05	−1.92	0.11	−4.86	0.05	2.22	0.11	−1.64	0.09	4.22	0.11	0.86	0.11	6.40	0.00
2	−8.22	0.19	−0.58	0.23	−5.60	0.19	3.32	0.04	−2.48	0.28	5.78	0.15	−0.28	0.11	8.38	0.18
3.2	−9.46	0.21	1.68	0.25	−6.48	0.16	4.84	0.13	−4.00	0.00	8.54	0.22	−0.98	0.16	10.20	0.00
	C16:C18 (20:80)				C16:C18 (10:90)				C16:C18 (0:100)							
	300 g/l				300 g/l				300 g/l							
0.25	1.00	0.24	3.76	0.05	2.54	0.42	4.88	0.04	4.36	0.19	5.96					
1	0.18	0.13	4.86	0.11	1.93	0.17	6.08	0.11	3.06	0.51	7.04					
2	−0.20	0.42	6.40	0.37	0.73	0.48	8.14	0.22	2.58	0.60	8.94					
3.2	−1.48	0.19	9.16	0.22	0.24	0.34	10.92	0.45	1.90	0.62	11.54					
	350 g/l				350 g/l				350 g/l							
0.25	2.48	0.25	4.90	0.00	3.58	0.22	6.00	0.00	5.14	0.34	7.16					
1	1.64	0.21	6.32	0.11	3.00	0.25	7.72	0.15	4.50	0.31	8.78					
2	1.18	0.31	7.76	0.21	2.60	0.12	10.32	0.15	3.62	0.51	10.16					
3.2	−0.12	0.36	10.20	0.41	1.92	0.22	12.08	0.49	3.12	0.23	13.02					
	400 g/l				400 g/l				400 g/l							
0.25	3.16	0.25	5.80	0.00	4.54	0.27	7.06	0.05	6.38	0.38	8.14					
1	2.52	0.22	7.08	0.11	4.20	0.12	8.54	0.15	5.20	0.58	9.86	0.17				
2	2.06	0.38	8.64	0.25	3.08	0.43	10.62	0.23	5.32	0.51	11.50	0.10				
3.2	1.02	0.24	11.64	0.09	2.96	0.28	13.50	0.17	4.50	0.21	13.82	0.32				
	450 g/l				450 g/l				450 g/l							
0.25	4.15	0.19	6.64	0.09	5.53	0.26	7.84	0.05	7.20	0.17	9.02	0.04				
1	2.82	0.26	8.20	0.00	4.86	0.48	9.34	0.15	6.18	0.61	10.74	0.09				
2	2.38	0.34	10.24	0.15	4.13	0.43	12.00	0.16	6.08	0.13	13.14	0.11				
3.2	1.94	0.25	12.28	0.13	3.22	0.13	14.02	0.33	5.08	0.26	15.36	0.25				

**Fig. 1:**
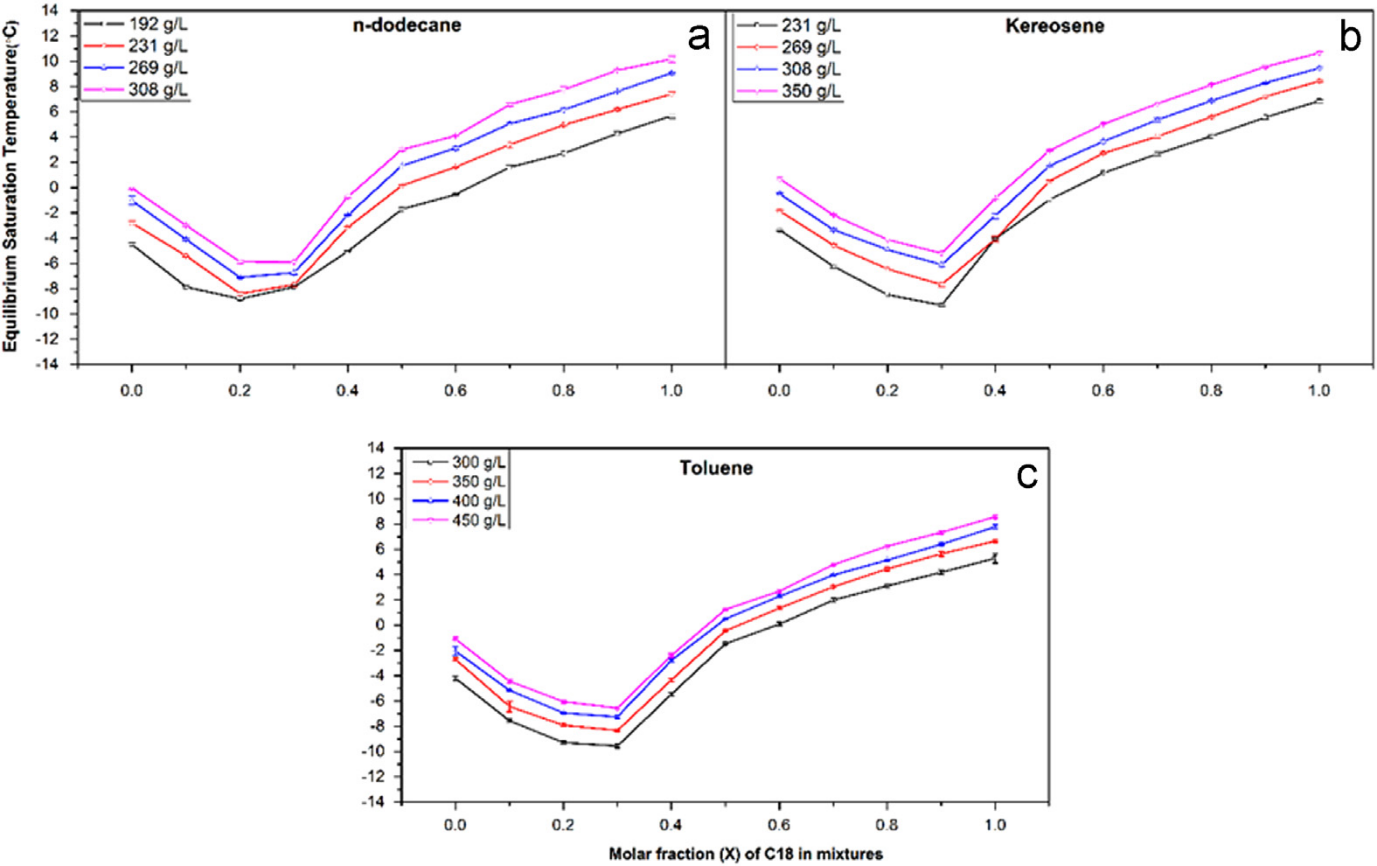
Equilibrium saturation temperature patterns as a function of C_16_H_34_/C_18_H_38_ mixtures in three solvents of (a) dodecane in concentrations of (192 g/l to 308 g/l); (b) kerosene in concentrations of (231 g/l to 350 g/l) and (c) toluene (300 g/l to 450 g/l. The data highlights that the shape of the solubility profile is not broadly dependent on either the solute concentration or the solvent type.

Fig. 1 shows the equilibrium saturation temperature (*T*_e_) patterns as a function of C_16_H_34_/C_18_H_38_ mixtures in the three solvents dodecane, kerosene and toluene over the range of compositions used and solution concentrations. Similar trends are seen over the range of solvents used with regards to the changing *T*_e_ values for different compositions and concentrations. The data highlights that the shape of the solubility profile is broadly independent of both the solvent type (aromatic, aliphatic or a mixture) and the solute concentration.

## 2 Experimental design, materials, and methods

### 2.1 Materials

Model compounds present in petrodiesel and the main constituents of HVOs were chosen for this study: hexadecane and octadecane, which were chosen alongside solvents that are representative of a diesel fuel mixture, in this case an aliphatic (dodecane), aromatic (toluene) and a mixture of the two cases (kerosene), see Table 4, Table 5.

**Table 4:** Summary of Materials used for experimental research.

**Chemical Name**	**Formula**	**Synonym**	**Purity (%)**	**MWg/mol**	**Supplier**
n-Hexadecane	C_16_H_34_	C16	≥99	226.44	Sigma–Aldrich
n-Octadecane	C_18_H_38_	C18	99	254.49	Sigma–Aldrich
n-dodecane	C_12_H_26_	C12	99	170.33	Sigma–Aldrich
Toluene	C_7_H_8_	N.A.	99	92.14	Sigma–Aldrich
Kerosene	N.A.	N.A.	99	167.90	Infineum Ltd.

**Table 5:** Composition information of kerosene as supplied by Infineum Ltd.

**Molecular type**	**wt%**
Paraffins	39.33
Naphthenes	42.41
Alkyl benzenes	7.62
Benzocycloparaffins	6.79
Naphthalenes	3.43
Biphenyls/acenaphthenes	0.27
Fluorenes	0.15
Phenanthrenes	0.00

### 2.2 Equipment

The crystallisation experiments were carried out using the Technobis Crystal 16® system [1]. This system comprises of sixteen wells that are split into four blocks, consisting of four wells each. Each block is independently temperature controlled to allow different temperature profiles to be set simultaneously, with the use of Peltier elements and an external cooling device. The system can be run between −15 °C to 100 °C and condensation at cooler temperatures is stopped by a dry air purge system. Each well can hold a magnetically stirred 1 ml disposable glass vial. A laser passes through the vials in order to determine turbidity and provide information about the cloud and clear points of up to 16 solutions and turbidity data is recorded as a function of the temperature.

### 2.3 Experimental procedure

#### 2.3.1 Solution preparation

All solutions were prepared on a 5 ml scale, enabling a sufficient amount of solution to be created for use in a single block of the Technobis Crystal 16® system. 11 binary mixed samples of differing molar ratios varying from 0% of C_18_H_38_ in each C_16_H_34_/C_18_H_38_ sample were first prepared. For each composition of the C_16_H_34_/C_18_H_38_ mixture, the solutions were made in four concentrations (192, 231, 269, 308 g/l), (231, 269, 308, 350 g/l) and (300, 350, 400, 450 g/l) for solvents of dodecane, kerosene and toluene, respectively. Each sample was weighed using a balance with ±0.1 mg accuracy.

Next, the mixtures were placed in a shaker with an external temperature control system (F32 Julabo temperature bath), at 55 °C for 30 min, with no shaking, in orderto form a melt. After this, a Fisherbrand 100–1000 μl micropipette was used to add 5 ml of toluene to each concentration sample. The resulting mixtures were placed in the same shaker, at 50 °C for 30 min under a shaking rate of 150 rpm, in order to form a homogeneous liquid solution. Next, for each concentration 4 Technobis Crystal 16® 1 ml vials were filled with 1 ml of the solution using a Fisherbrand 100–1000 μl micropipette and a standard small magnetic stirrer was added to each vial. This resulted in one composition forming sixteen 1 ml glass vial samples, with four vials containing one of the set concentrations.

#### 2.3.2 Measurement and analysis

Each vial contained a magnetic stirrer, which was set to stir at 700 rpm for all samples analysed. Each block contained the four concentrations to be analysed, as described previously, meaning one cooling/heating rate (q) would be used over the entire range of concentrations for each solution composition. Different temperature cycles were set up for each block in the unit using external temperature control and the Crystal16 computer software, and due to the high amounts of data recorded by the system, the cooling/heating rates (q) of 1 °C/min, 2 °C/min and 3.2 °C/min were run initially and upon their completion the cooling/heating rate of 0.25 °C/min was run. Each temperature cycle began by heating the solutions to 40 °C, with this temperature being held for one hour to ensure that complete homogenization had taken place and were then subsequently cooled at a set rate, as aforementioned, to −15 °C. This temperature was then held for an hour to allow equilibration, followed by an increase in temperature back to 40 °C at the specified rate. Each temperature cycle was performed five times to obtain average values for the crystallisation and dissolution temperatures. This enabled data fitting improvement, whilst also reducing the standard deviation (std) of the measured temperatures.

Once the unit had run the cooling rates over the range of concentrations for a solution composition, data on turbidity was collected in the form of the relationship between the transmission of a laser through the sample and the temperature of the sample. Crystallisation (*T*_c_) and dissolution (*T*_diss_) temperatures were estimated from turbidity data, which ranged from 0% for a fully crystallised solution and 100% for a homogeneous solution. *T*_c_ was taken as the point where light transmittance dropped by 10% from 100% and *T*_diss_ was taken at the point where light transmittance reached 100%. The collected *T*_c_(*q*) and *T*_diss_(*q*) data was used to form *T*_c_(*q*) and *T*_diss_(*q*) graphical lines that were extrapolated back to *q*=0 °C/min in order to determine equilibrium saturation temperatures (*T*_e_).
