# Supplementary material for: Data for crystallisation, dissolution and saturation temperatures of the ternary system: Hexadecane and octadecane representative in fuel solvents
Source: Data Brief. 2018 May 31;19:1382–92. doi: 10.1016/j.dib.2018.05.136 (PMC6141768; doi:10.1016/j.dib.2018.05.136)
Supplement: Supplementary file 1 — Supplementary material [file mmc1.docx]

**Conflict of Interest Form**

The authors declare no conflict of interest associated with this publication.
